# SGLT2 Inhibitors Confer Cardiovascular Protection via the Gut-Kidney-Heart Axis: Mechanisms and Translational Perspectives

**DOI:** 10.3390/jcdd12120471

**Published:** 2025-11-30

**Authors:** Yimei Tao, Ning Zhang, Zhaoxiang Wang, Ying Pan, Shao Zhong, Hongying Liu

**Affiliations:** 1 Department of Endocrinology, Gusu School, Nanjing Medical University, The First People’s Hospital of Kunshan, Kunshan 215300, China; 18168706847@163.com (Y.T.); drzhong@163.com (S.Z.); 2 Department of Endocrinology, Affiliated Kunshan Hospital of Jiangsu University, Kunshan 215300, China; 15720758834@163.com (N.Z.); wzx19970101@163.com (Z.W.); 3 Department of General Practice, Affiliated Kunshan Hospital of Jiangsu University, Kunshan 215300, China; py97183@163.com; 4 Hangzhou Kang Ming Information Technology Co., Ltd., Hangzhou 310000, China

**Keywords:** SGLT2 inhibitors, gut-kidney-heart axis, gut microbiota, cardiovascular protection, metabolic reprogramming

## Abstract

Sodium-glucose cotransporter 2 inhibitors (SGLT2i) have demonstrated significant cardiovascular and renal benefits beyond glycemic control, yet their integrated mechanisms remain incompletely understood. Emerging evidence highlights the gut-kidney-heart axis as a pivotal pathological network, wherein gut dysbiosis, toxic metabolite accumulation, intestinal barrier disruption, and systemic inflammation synergistically drive cardiorenal injury. This review systematically elucidates how SGLT2i modulate this axis through multi-level interventions: reshaping gut microbiota composition, enriching short-chain fatty acid-producing bacteria, suppressing trimethylamine and other toxin-generating microbes, restoring tight junction integrity, and regulating bile acid metabolism. These upstream effects reduce systemic inflammatory and metabolic stress, interrupt kidney-derived toxin amplification, and mitigate myocardial remodeling. Unlike previous reviews focusing on single-organ pathways, this work integrates microecological regulation, metabolite reprogramming, and cross-organ protection into a unified “three-axis convergence to the heart” framework. We also highlight potential species-specific microbiota regulatory profiles among different SGLT2i and propose future directions, including fecal microbiota transplantation and microbiota-targeted co-therapies, to clarify causal relationships and optimize therapeutic strategies. By positioning the gut as a modifiable upstream driver, this framework provides novel mechanistic insight and translational potential for expanding SGLT2i applications in metabolic cardiovascular disease, including in non-diabetic populations.

## 1. Introduction

Sodium-glucose cotransporter 2 inhibitors (SGLT2i) have attracted wide attention for their significant benefits in cardiovascular and renal outcomes. SGLT2i were originally developed to reduce glucose reabsorption in the proximal renal tubules. However, recent studies show that their benefits in heart failure, chronic kidney disease, and cardiovascular events are not entirely dependent on diabetes status [1]. This suggests that the protective effects of SGLT2i may go beyond their traditional glucose-lowering mechanisms, such as osmotic diuresis, reduced cardiac preload, and improved energy metabolism. They may also act through a range of non-glycemic mechanisms to protect target organs [2,3,4].

## 2. Pathological Foundations of the Gut-Kidney-Heart Axis

**Figure 1:**
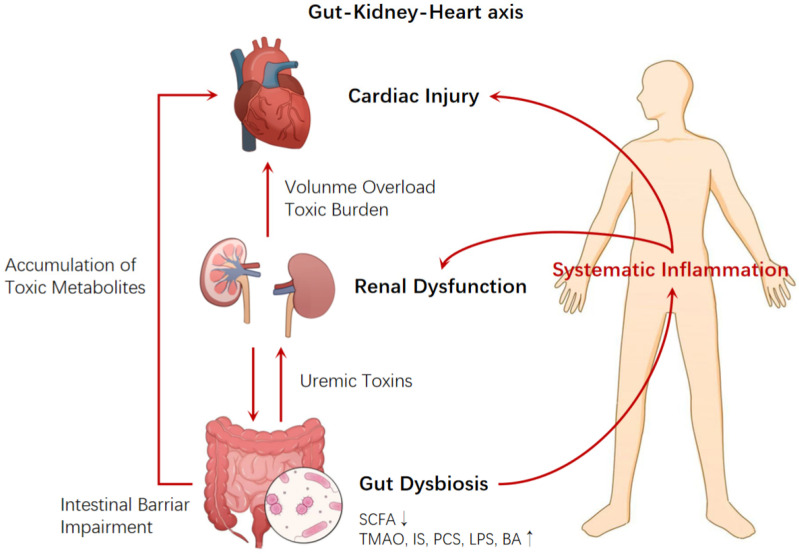
The gut-kidney-heart axis, illustrating the interaction between gut microbiota dysbiosis, renal dysfunction and cardiac injury. This interaction involves the accumulation of metabolites, inflammatory responses and systemic effects.

The gut microbiota, as a key “remote organ” in host metabolism and immune regulation, plays an upstream role in the development of heart and kidney diseases. A growing body of research indicates that disruptions in gut microbial composition can influence distant organs through microbial-derived metabolites [5,6,7]. This creates critical links in both the “gut-heart axis” and the “gut-kidney axis.” This section focuses on how microbial metabolites contribute to cardiac and renal dysfunction through inflammation, toxic burden, and metabolic disorders, thereby laying the foundation for understanding SGLT2i interventions (Figure 1) [8].

### 2.1. Gut Dysbiosis: The Upstream Origin

The gut microbiota is the most complex and metabolically active symbiotic ecosystem in the human body, consisting of bacteria, fungi, archaea, protozoa, and viruses. Over the course of evolution, this community has developed a close, mutually beneficial relationship with the host [9,10]. It supports immune development, helps maintain metabolic balance, and contributes to neuroendocrine regulation [11]. In a healthy state, the microbiota remains diverse and relatively stable [12]. Beneficial microbes produce short-chain fatty acids (SCFAs), reinforce the intestinal barrier, modulate inflammatory pathways, and help protect against pathogenic organisms, actions that extend their influence far beyond the gut itself [13]. Once this equilibrium is disrupted, however, changes in microbial composition and function may occur, and such dysbiosis has been linked to a wide range of chronic conditions, including obesity, diabetes, cancer, and neurodegenerative diseases [14].

However, this balance can be disturbed by many internal and external factors. Among them, diet remains one of the most decisive determinants. Western-style diets, high in fat and sugar but low in fiber, consistently reduce microbial diversity, suppress SCFA-producing bacteria, and favor the growth of species that generate harmful metabolites [15,16]. As a result, the metabolic landscape of the gut begins to shift. Lifestyle habits also matter. Physical inactivity, long periods of sitting, and ongoing psychological stress can influence gut motility, immune activity, and barrier function, gradually driving the system toward dysbiosis. The metabolic disorders that follow, such as obesity and insulin resistance, further intensify local inflammation and alter the intestinal environment, creating a self-reinforcing cycle. Medications add another layer of disruption. Antibiotics, proton pump inhibitors, and some antidiabetic drugs can reshape the gut ecosystem by altering pH levels, changing nutrient availability, or directly reducing commensal populations [17].

Importantly, in obesity, inflammatory cytokines released from adipose tissue (such as TNF-α and IL-6) drive chronic low-grade inflammation and compromise the integrity of the intestinal barrier, allowing endotoxins to enter the systemic circulation and contributing to gut dysbiosis [18]. In type 2 diabetes, hyperglycemia and metabolic inflammation further reshape the gut ecosystem. Excess luminal glucose supports the growth of opportunistic pathogens, while impaired mucosal immunity and oxidative stress make it difficult for beneficial commensal species to persist [19,20,21]. Prolonged insulin resistance also affects gut motility, enteroendocrine signaling, and bile-acid composition, promoting bacterial overgrowth and mucosal inflammation and further disturbing microbial homeostasis [22]. Glucose-lowering treatments such as metformin can, to some extent, help restore microbial balance [23]. Taken together, metabolic disturbances are not only a consequence of gut dysbiosis but also an important contributor to its development.

### 2.2. The Closed-Loop Pathophysiology of the Gut-Kidney-Heart Axis

First, gut dysbiosis leads to structural damage of the intestinal barrier. This results in reduced expression of tight junction proteins (TJPs) and mucus layer components (such as MUC2), causing increased intestinal permeability, also known as “leaky gut.” As a result, harmful metabolites such as lipopolysaccharide (LPS) and trimethylamine-N-oxide (TMAO) enter the circulation.

Once gut-derived metabolites enter the bloodstream, they may impair kidney function by inducing inflammation, oxidative stress, and tissue fibrosis. In chronic kidney disease (CKD), patients often exhibit increased gut permeability, elevated inflammatory markers, and reduced production of short-chain fatty acids (SCFAs). Declining renal clearance leads to the accumulation of protein-bound uremic toxins, such as indoxyl sulfate and p-cresyl sulfate [24]. These toxins can directly damage both the kidneys and cardiovascular system. They also worsen gut dysbiosis and barrier dysfunction, creating a vicious cycle [25,26]. This cycle amplifies kidney injury and microbial imbalance, which in turn increases cardiovascular burden and promotes inflammatory signaling to the heart [6].

Finally, the heart becomes one of the most severely affected organs, ultimately leading to myocardial fibrosis and structural remodeling [27,28]. CKD itself is a major risk factor for cardiovascular events. CKD-related volume overload, endothelial dysfunction, and sympathetic activation, together with gut-derived toxins, accelerate the development of heart failure [29]. Studies show elevated blood levels of LPS and TMAO in both CKD and heart failure patients, which positively correlate with NT-proBNP, hs-CRP, and cardiac remodeling markers [5]. This suggests that gut metabolites may be key drivers behind cardiac structural and functional changes.

In summary, the gut-kidney-heart axis forms a typical inflammatory toxic metabolic feedback loop, driven by the sequential process of dysbiosis, toxin production and retention, systemic inflammation, and organ injury. This closed-loop model not only implicates multiple organs but also offers a new perspective: Can we break this vicious cycle by targeting the gut microbiota at the upstream source? While reducing circulating toxins is important, a more critical question is how to prevent the production and absorption of these toxins and their precursors. Maintaining a balance between harmful and beneficial metabolites is essential. Since the gut microbiota is the major source of many uremic and inflammatory toxins, understanding how to regulate this axis, especially by restoring microbial balance, repairing the gut barrier, and blocking the gut-kidney amplification loop, may open a new path toward cardiovascular protection.

## 3. Gut Initiation: Microbiota Remodeling and Systemic Modulation by SGLT2i

As described above, gut dysbiosis is one of the upstream drivers in the gut-kidney-heart pathological network. Overall, it creates a vulnerable internal environment through a set of shared non-specific mechanisms, which provides the basis for progressive injury to both the kidneys and the heart.

### 3.1. Microbiota-Derived Non-Specific Systemic Injury

First, in a dysbiotic state, levels of harmful gut-derived metabolites such as TMAO, LPS, indoxyl sulfate (IS), and p-cresyl sulfate (PCS) are significantly elevated. Once these molecules pass through the compromised gut barrier into circulation, they can trigger systemic damage through various mechanisms. For instance, LPS activates the Toll-like receptor 4 (TLR4) pathway, leading to NF-κB-dependent proinflammatory cytokine release and persistent low-grade inflammation [30]. TMAO promotes oxidative stress, impairs mitochondrial function, and disrupts lipid metabolism, resulting in oxidative injury and apoptosis in multiple tissues [31]. Moreover, many of these toxins suppress the expression of tight junction proteins (e.g., ZO-1, Occludin), further damaging the gut barrier and worsening systemic exposure to endotoxins and inflammatory signals [32]. Chronic exposure to these toxins may also activate immune sensing pathways such as the aryl hydrocarbon receptor (AhR), alter cytokine transcription, and skew immune cell polarization, ultimately disrupting immune homeostasis [33]. These mechanisms have been implicated in various systemic disorders, suggesting that gut dysbiosis and its metabolites are key drivers of metabolic and inflammatory diseases.

Second, the depletion of “beneficial metabolites” is equally important. SCFAs such as acetate, propionate, and butyrate are produced by anaerobic fermentation. They help maintain gut barrier integrity, stimulate GLP-1 and PYY secretion to improve glucose and lipid metabolism, regulate immune responses via TLR4/NF-κB, and inhibit inflammation by activating GPCRs or suppressing histone deacetylases (HDAC). SCFAs also reduce monocyte infiltration and fibrosis-associated signaling, offering anti-inflammatory and antioxidant effects. These functions are protective against cardiovascular and kidney diseases [34,35,36]. In patients with renal impairment, SCFA-producing bacteria are reduced, and butyrate levels are significantly decreased. This indicates that microbial dysfunction not only weakens the gut barrier but also impairs anti-inflammatory defenses, contributing to uremic toxin accumulation and creating a vicious cycle of microbial-immune-barrier imbalance—a so-called “gut-derived inflammatory storm.”

Beyond systemic injury through inflammation, oxidative stress, and barrier disruption, gut dysbiosis and its metabolites can also exert organ-specific effects. Different toxins show preferential distribution and targeting. For example, TMAO mainly affects cardiac metabolism and electrical stability, while IS and PCS are more involved in kidney injury and interstitial fibrosis. Gut microbiota can also influence bile acid metabolism and host miRNA expression, promoting endothelial dysfunction and ventricular remodeling. These targeted injury mechanisms will be further discussed in Section 4 and Section 5, focusing on the kidneys and the heart, respectively.

On this shared susceptibility platform, SGLT2 inhibitors (SGLT2i) demonstrate multiple regulatory potentials. Increasing evidence shows that SGLT2i not only lower glucose and promote natriuresis but also reshape gut microbial composition, restore barrier function, and modulate inflammatory pathways. These actions may block key links in the cascade of non-specific systemic injury. By re-establishing metabolic and immune homeostasis, SGLT2i lay the groundwork for downstream organ-specific protection.

### 3.2. Microbiota Remodeling by SGLT2i as a Mechanism to Reduce Systemic Injury

#### 3.2.1. Reshaping Microbial Composition: Increasing SCFAs and Reducing Harmful Metabolites

Animal studies demonstrate that SGLT2i (e.g., empagliflozin, dapagliflozin, canagliflozin) modulate gut microbiota by enriching SCFA-producing genera (e.g., *Bifidobacterium*, *Bacteroides*) and suppressing pathogens (e.g., *Clostridium*, *Shigella*). These changes are accompanied by elevated butyrate levels, reduced lipopolysaccharide (LPS) concentrations, and improved inflammatory profiles [37,38]. Similar results have been observed in models of type 1 diabetes, chronic kidney disease (CKD), diabetic nephropathy, non-diabetic, and obesity-related glomerulopathy [39].

Importantly, a causal relationship has been supported by fecal microbiota transplantation (FMT) studies. Transplanting gut microbiota from mice treated with phlorizin (PHZ, a compound similar to SGLT2i) into untreated mice reproduced improvements in metabolic disorders and intestinal barrier integrity in the recipients [40]. In an atherosclerosis model using ApoE-knockout mice, empagliflozin significantly increased beneficial microbial taxa such as *Aromaticibacter*, *Clostridiaceae*, and the S24-7 group, thereby mitigating disease progression. FMT from empagliflozin-treated mice into high-fat diet (HFD)-fed ApoE^−/−^ mice also reproduced atherosclerosis attenuation and reduced inflammation, confirming a microbiota-dependent protective mechanism [41].

Human studies show a similar trend. In patients with diabetes, an 8-week treatment with dapagliflozin significantly increased the abundance of butyrate-producing bacteria (e.g., *Butyricicoccus*), along with reductions in C-reactive protein (CRP) and fecal LPS levels. A 2022 study in type 2 diabetes patients, with medication variables controlled, demonstrated that empagliflozin enhanced the abundance of SCFA-producing species (e.g., *Roseburia*, *Eubacterium*, *Faecalibacterium*) while reducing harmful species like *Shigella*, *Desulfovibrio*, and *Hungatella* [10]. Notably, even short-term (2-week) canagliflozin treatment decreased levels of gut-derived nephrotoxins (e.g., PCS and IS) and increased SCFA concentrations [42]. Although these beneficial shifts in gut microbiota are evident, current human studies have yet to establish definitive causal links (e.g., through FMT trials).

#### 3.2.2. Enhancing Gut Barrier Function and Alleviating Endotoxin-Driven Inflammation

Beyond modulating microbial composition, SGLT2i also improve gut barrier integrity by upregulating tight junction proteins in the intestinal epithelium. This reduces the translocation of endotoxins such as LPS into systemic circulation, thereby exerting anti-inflammatory and cardiovascular protective effects.

In diabetic and obese mouse models, dapagliflozin significantly increased the expression of tight junction proteins ZO-1, Occludin, and Claudin in colonic epithelial cells. It also enhanced goblet cell secretion of MUC2 mucin, thereby restoring mucosal architecture. Similarly, in diabetic rats, downregulation of Zonulin-1 and Occludin was reversed by empagliflozin treatment [40], and dapagliflozin was also observed to upregulate Occludin and Claudin expression.

These modifications reduce the risk of “leaky gut,” limiting LPS-induced systemic inflammation and indirectly alleviating endothelial dysfunction [39]. Furthermore, SGLT2i increase butyrate-producing bacteria such as *Bifidobacterium*, *Faecalibacterium*, and *Butyricicoccus*, which are closely associated with tight junction protein expression. This suggests that the protective effects of SGLT2i may result from the synergistic interaction of “microbiota–metabolites–barrier” pathways.

Although these microbiota shifts and SCFA increases are clearly linked to metabolic benefits, the underlying molecular mechanisms remain to be fully elucidated.

#### 3.2.3. Species-Specific Microbial Modulation by SGLT2i

Notably, different types of sodium-glucose co-transporter 2 inhibitors (SGLT2i) may exert specific regulatory effects on the gut microbiota. For example, in diabetic mouse models, canagliflozin not only promotes SCFA production but also enhances glucagon-like peptide-1 (GLP-1) secretion and regulates bile acid metabolism. After treatment, beneficial microbes such as *Muribaculum*, *Ruminococcaceae*_UCG_014, and *Lachnospiraceae*_UCG_001 were significantly increased. Meanwhile, levels of secondary bile acids, such as ursodeoxycholic acid (UDCA) and hyodeoxycholic acid (HDCA), were reduced. These changes were closely related to improved fatty acid metabolism and insulin sensitivity [43]. In addition, Yang et al. found that dapagliflozin significantly decreased the abundance of *Fusobacterium nucleatum* [44]. This bacterium converts hydrophobic bile acids into glycoconjugated bile acids. These metabolites activate endothelial bile acid receptors and induce endothelial dysfunction [45,46]. This process is a key contributor to atherosclerosis in type 2 diabetes [47,48,49].

Although existing studies suggest that SGLT2i may have species-specific effects on gut microbiota, these effects may also depend on dosage. The same SGLT2i at different doses may cause variations in microbial composition and metabolite levels. However, systematic comparisons of different SGLT2i under controlled variables (e.g., dosage, treatment duration, diet) are still lacking. Future research should explore how SGLT2i type and dose affect gut microbiota and related metabolic pathways. This will provide a solid theoretical and experimental basis for personalized and targeted interventions in metabolic diseases.

### 3.3. Potential Mechanisms Behind SGLT2i-Induced Microbiota Changes

Although the exact mechanisms remain unclear, some studies have provided preliminary insights into the gut-level actions of SGLT2i. These drugs may partially inhibit SGLT1 activity in the intestine. In human studies, a 300 mg dose of canagliflozin transiently inhibited intestinal SGLT1 function and reduced glucose absorption in the small intestine [50]. This inhibition allows more glucose to reach the distal gut, including the ileum and colon [51]. The redistribution of glucose absorption increases colonic glucose fermentation. This shift affects the metabolic activity and composition of the gut microbiota. Therefore, SGLT2i may participate in metabolic regulation through direct actions in the gut.

In summary, SGLT2i may protect multiple organs by reshaping the gut microbiota, increasing SCFA production, and enhancing gut barrier function. Although causal evidence is still limited, these mechanisms offer a new explanation for their cardio-renal protective effects beyond glucose control. They also suggest a possible intervention point within the gut-kidney-heart axis.

Microbial-derived metabolites have been linked to multi-organ damage involving the heart and kidneys. These metabolites have upstream driving effects and systemic amplification properties, providing a theoretical basis for early intervention. Notably, their toxicity is tissue-specific: TMAO mainly targets the heart, while IS and PCS primarily damage the kidney and skeletal muscle. These findings suggest that modulating gut microbiota, reducing toxic metabolite production, and restoring barrier function may block the harmful cascade from the source. This could provide a critical entry point for future therapeutic strategies.

## 4. Renal Hub: How SGLT2 Inhibitors Disrupt the Gut-Kidney-Heart Loop

In the gut-kidney-heart axis, the kidney is not only a target organ of damage but also a critical node for toxin clearance, immune regulation, and metabolic homeostasis. Uremic toxins related to kidney function can be categorized into three types based on their origin: endogenous (produced by host cell metabolism), exogenous (mainly from diet), and microbial-derived (formed by gut microbiota metabolism). Among these, microbial-derived toxins play a crucial role in the onset and progression of chronic kidney disease (CKD) [52].

As mentioned earlier, the gut microbiota and kidney form a gut-kidney axis. Their relationship is not a simple unidirectional causality. Instead, they engage in a close and complex bidirectional pathological dialogue. They influence and promote each other, creating a vicious cycle. SGLT2 inhibitors may disrupt this loop through multidimensional regulation.

### 4.1. Gut Microbiota Metabolites Mediating “Gut-Derived Kidney Injury”

Gut dysbiosis is recognized as an important trigger for CKD progression. Its pathogenicity is driven by systemic damage, including increased generation of harmful metabolites, reduced beneficial metabolites, and impaired gut barrier function. These factors synergistically exacerbate renal structure and functional deterioration, along with organ-specific injury [53,54].

Firstly, in states of abnormal microbial composition, protein fermentation products such as indoxyl sulfate (IS), p-cresyl sulfate (PCS), and trimethylamine-N-oxide (TMAO) accumulate in the body [55,56,57]. These protein-bound uremic toxins (PBUTs) are difficult to remove via dialysis. They enter renal tubular epithelial cells through organic anion transporters (OATs) and activate signaling pathways including aryl hydrocarbon receptor (AhR) and nuclear factor kappa B (NF-κB). This causes podocyte injury, characterized by reduced viability and morphological changes, as well as glomerular damage. It induces expression of pro-inflammatory cytokines such as TNF-α, IL-6, and MCP-1 and promotes TGF-β-mediated interstitial fibrosis [58,59]. Moreover, IS triggers mitochondrial dysfunction, increases reactive oxygen species (ROS) production, and inhibits antioxidant pathways such as Nrf2 [60]. These effects further induce oxidative stress and apoptosis. Beyond these mechanisms, TMAO regulates renal vascular tone via endothelial ET-1/AngII systems. It accelerates glomerulosclerosis under hyperperfusion and hyperfiltration states and elevates systemic blood pressure, contributing to cardiovascular damage [61]. Persistent accumulation of these toxins also downregulates OAT expression, impairing toxin clearance and exacerbating renal injury, thus forming a vicious cycle [62].

Simultaneously, dysbiosis reduces SCFA-producing beneficial bacteria, causing systemic SCFA deficiency. SCFAs provide essential energy substrates for renal tubules and exert multiple protective effects by regulating epigenetics, immune homeostasis, and antioxidant systems to maintain kidney function [63]. On one hand, SCFAs promote mitochondrial metabolism in renal tubular epithelial cells, maintaining ATP and NAD^+^ levels, reducing mitochondrial fragmentation and energy crises. This delays progression from acute kidney injury to CKD [64]. On the other hand, butyrate, a natural histone deacetylase (HDAC) inhibitor, suppresses pro-inflammatory and pro-fibrotic gene expression and enhances Nrf2 antioxidant pathway activity, counteracting toxin-induced inflammation [65]. Furthermore, SCFAs activate G-protein-coupled receptors (GPR41/43), modulating renal macrophage M1/M2 polarization, maintaining Treg/Th17 balance, and inhibiting NLRP3 inflammasome activation. These effects suppress renal inflammatory cascades [66]. Importantly, SCFAs stabilize hypoxia-inducible factor 1-alpha (HIF-1α), enhancing renal tolerance to hypoxic stress, promoting angiogenesis, and metabolic adaptation [67].

Ultimately, through impaired gut barrier function, the accumulation of gut-derived toxins, energy deficit, and dysregulated inflammatory responses collectively drive tubular epithelial injury, glomerulosclerosis, and interstitial fibrosis. This promotes CKD progression and markedly increases cardiovascular risk by affecting systemic metabolism and vascular function.

### 4.2. SGLT2 Inhibitors Modulate Kidney Injury via the “Gut-Microbiota-Metabolite” Axis

Considering the multiple impacts of gut-derived toxic metabolites and SCFA deficiency on kidney injury, SGLT2 inhibitors (SGLT2i) have been found to alleviate this process by regulating gut microbiota and their metabolites at various levels. Prospective clinical trials show that dapagliflozin significantly improves renal outcomes early in treatment. Its renoprotective effect persists during long-term follow-up [68]. This suggests that mechanisms beyond glucose lowering, such as systemic remodeling of the gut microbiome, may be involved.

Animal studies further reveal that SGLT2i intervention alters gut microbial composition and markedly affects microbial metabolite profiles, thereby reducing renal burden at multiple points. For example, mice fed a high-salt diet (HSD) exhibit gut dysbiosis with elevated serum levels of toxic metabolites including methylhistidine, creatinine, homoarginine, and indoxyl sulfate. This reflects enhanced microbial protein fermentation and closely correlates with chronic kidney dysfunction [69,70,71]. Canagliflozin treatment effectively reconstructs gut microbial ecology, reducing systemic accumulation of these metabolites and mitigating HSD-induced glomerular dysfunction and oxidative stress. Improvements in serum creatinine and superoxide dismutase (SOD) levels support these findings [72]. Similarly, other studies report that canagliflozin reduces kidney-toxic metabolites such as PCS and IS within 2 weeks of treatment [42,73].

Similar results are observed in diabetic nephropathy (DN) models. Ni et al. reported that low-dose dapagliflozin effectively ameliorated renal tissue injury induced by high-fat diet combined with streptozotocin (STZ), without significantly altering blood glucose. Manifestations included reduced proteinuria and alleviated inflammation and fibrosis. Mechanistically, this effect was accompanied by increased abundance of beneficial gut bacteria, such as *Bifidobacterium*, *Prevotellaceae*, and *Muribaculaceae*, along with decreased pathogenic bacteria associated with uremic toxin production, including *Desulfovibrionaceae* and *Clostridiaceae*. The reshaped microbiota drove metabolic reprogramming, promoting the production of SCFAs like succinic acid (SAC), acetate, and butyrate, which exert anti-inflammatory, antioxidant, and barrier repair effects. Simultaneously, it suppressed accumulation of pro-inflammatory and pro-fibrotic toxins such as ASA, phenylacetic acid (PA), and lipopolysaccharide (LPS). In vitro studies confirmed that ASA and PA directly damage renal tubular epithelial cells, whereas SAC, a key sulfur metabolism intermediate elevated by dapagliflozin, showed kidney-protective activity resembling drugs, indicating its possible role as a downstream mediator. Notably, broad-spectrum antibiotic treatment that depleted gut microbiota markedly attenuated these protective effects, underscoring the critical role of gut ecology in dapagliflozin-mediated renoprotection [37].

Similar findings were reported in other models, such as obesity-related glomerulopathy (ORG) [74] and diabetic nephropathy. Moreover, as mentioned previously, besides altering microbial composition, SGLT2i significantly upregulate tight junction proteins ZO-1, Occludin, and Claudin in colonic epithelial cells, and enhance goblet cell secretion of mucin MUC2, effectively restoring gut mucosal integrity [40]. This reduces systemic inflammation induced by harmful substances like LPS, indirectly alleviating endothelial dysfunction [39].

In summary, the benefits of SGLT2i result from the synergistic effects of microbiota, metabolites, and barrier function. These gut metabolic changes largely occur independently of glucose control. Overall, dysbiosis caused by various etiologies can directly or indirectly induce renal dysfunction. SGLT2i regulate gut ecology, metabolite profiles, and barrier integrity to potentially interrupt the gut-kidney axis. This provides a mechanistically clear and comprehensive intervention strategy for CKD.

### 4.3. Gut Microbiota Dysbiosis Induced by Renal Dysfunction and Its Restoration by SGLT2i

Renal dysfunction also disrupts gut microbiota through multiple pathways. Studies show that impaired kidney function significantly alters gut microbial composition, including decreases in beneficial bacteria such as *Lactobacillaceae* and *Prevotellaceae*, alongside increases in *Enterococcus* and other bacteria [75].

Chronic kidney dysfunction is both a consequence and a cause of gut dysbiosis. As estimated glomerular filtration rate (eGFR) declines, uremic toxins including IS, PCS, urea, and creatinine accumulate in the body. Some small molecules diffuse into the intestinal lumen via mucosa, significantly altering gut physiology. This manifests as decreased luminal pH, elevated ammonia nitrogen, and osmotic imbalance [76]. Such a “uremic environment” is unfavorable to symbiotic bacteria, causing sharp reductions in SCFA producers such as *Faecalibacterium* and *Roseburia*, with concomitant increases in protein-fermenting toxin-producing bacteria like *Clostridium* and Enterobacteriaceae. This decreases microbial diversity and increases pathogenic bacteria proportion [77]. More seriously, renal dysfunction impairs intestinal barrier integrity, decreasing expression of tight junction proteins (e.g., ZO-1, Occludin), thinning the mucus layer, and increasing epithelial permeability. This facilitates translocation of bacterial products such as LPS into circulation, triggering systemic inflammation and immune activation, further damaging renal function [78].

Additionally, hyperuricemia caused by renal impairment directly influences gut microbiota composition. Elevated uric acid induces decreases in beneficial bacteria like *Bifidobacterium* and *Lactobacillus* and promotes proliferation of pro-inflammatory genera such as *Escherichia–Shigella*. This disrupts intestinal metabolic homeostasis, worsening systemic inflammation and barrier damage. These changes exacerbate downregulation of tight junction proteins, mucus layer thinning, and epithelial permeability elevation, increasing bacterial product translocation and activating systemic immune responses. This creates a positive feedback loop among kidney dysfunction, uric acid, and gut dysbiosis.

SGLT2 inhibitors reverse this dysbiosis via multiple mechanisms. First, they improve tubular metabolic state and reduce toxin load, effectively decreasing intestinal accumulation of toxic molecules and blocking the upstream source [79]. Second, SGLT2i promote uric acid excretion, improving abnormal systemic and intestinal uric acid accumulation. Studies show that although SGLT2i do not directly target urate transporters URAT1, GLUT9 isoform 1, or OAT4, they increase luminal glucose concentration in renal tubules, indirectly activating GLUT9 isoform 2 and inhibiting uric acid reabsorption, thus enhancing uric acid clearance. As systemic uric acid levels decline, the gut inflammatory environment associated with hyperuricemia is alleviated, allowing restoration of SCFA-producing bacteria and gradual microbiota balance recovery [80]. These findings indicate that SGLT2i act beyond traditional glucose metabolism and renal excretion pathways. They also repair gut ecological damage caused by renal dysfunction via a bottom-up mechanism by regulating uric acid metabolism. This breaks the pathological positive feedback loop of kidney-intestine inflammation and offers a novel therapeutic perspective for CKD patients.

## 5. Cardiac Endpoints: Heart-Specific Injury and Protective Mechanisms of SGLT2i

In the gut-heart and kidney-heart axes, SGLT2 inhibitors (SGLT2i) modulate gut microbiota, improve renal function, and metabolic status. This reduces toxin burden and systemic inflammation, indirectly alleviating cardiac structural and functional damage. This step serves as the integrated output and therapeutic target of the multi-axis protective mechanism.

### 5.1. Gut-Heart Axis and SGLT2i Regulatory Mechanisms

Apart from the non-specific mechanisms described above, accumulating evidence shows that the gut can damage the heart via certain cardiac-specific metabolites. These include trimethylamine-*N*-oxide (TMAO), bile acid metabolism products, and microRNAs. These factors activate a series of heart-specific injury mechanisms. These mechanisms extend beyond traditional inflammation or oxidative stress pathways, showing more targeted pathological effects [81,82].

Once LPS, TMAO, indoles, phenols, and cardiorenal toxic substances enter systemic circulation, they induce oxidative stress, trigger systemic inflammation, or directly damage cardiac and renal tissues. This markedly increases the risk of cardiorenal diseases. Especially regarding TMAO, accumulating evidence indicates it exerts distinct mechanisms beyond nonspecific inflammation and oxidative stress. TMAO is mainly derived from Western diets rich in phosphatidylcholine, choline, and carnitine. It is synthesized in the liver by flavin-containing monooxygenase 3 (FMO3), whose expression is regulated by the farnesoid X receptor (FXR) and bile acid levels [83]. TMAO generation depends jointly on diet, gut microbiota composition, and kidney function. Epidemiological studies link circulating TMAO levels closely with atherosclerosis [82], peripheral artery disease, coronary artery disease, acute coronary syndrome, and heart failure prognosis [84].

Notably, trimethylamine-*N*-oxide (TMAO) has been identified as a key gut-derived metabolite implicated in cardiac injury [85]. TMAO exacerbates myocardial damage through not only systemic inflammation but also direct cardiac-specific mechanisms. For instance, TMAO suppresses reverse cholesterol transport, impairs macrophage-mediated cholesterol efflux, and facilitates foam cell formation, thereby attenuating the protective effects of high-density lipoprotein (HDL) while inducing eNOS uncoupling, reducing NO bioavailability, and increasing reactive oxygen species (ROS) production, which collectively accelerate endothelial dysfunction and atherosclerotic plaque progression [86]. Additionally, TMAO enhances calcium-dependent platelet reactivity, elevating thrombotic risk. Beyond its vascular effects, TMAO permeates cardiomyocyte membranes to inhibit L-type calcium channels and disrupt the expression of calcium-regulating proteins (e.g., RyR2, SERCA2a), leading to calcium dyshomeostasis and ventricular arrhythmias. It also activates hypertrophic signaling pathways such as PI3K/Akt and MAPK, promoting cardiomyocyte hypertrophy and ventricular remodeling [87,88,89]. Furthermore, TMAO suppresses AMPK activity, shifting myocardial energy metabolism toward inefficient glycolysis and thereby aggravating heart failure [90].

Besides TMAO, gut microbiota also modulates host microRNA expression to participate in remote cardiac regulation. For instance, in animal studies, gut microbiota upregulate miR-146a expression in small intestinal epithelium via TLR4/TLR5–MyD88–NF-κB signaling [91]. Germ-free mice show reduced miR-223 levels, which normalize after microbial colonization [92]. Clinical data reveal plasma miR-146a-5p levels positively correlate with TMAO concentrations [93], indicating the microbiota-host co-metabolism system regulates cardiac pathology through miRNAs. Circulating miR-146a targets myocardial tissue, modulating contraction by inhibiting SERCA2a SUMOylation. Cardiac fibroblasts also transfer miR-146a to cardiomyocytes via exosomes, influencing intercellular communication, electrophysiological homeostasis, and ventricular remodeling.

Furthermore, gut dysbiosis affects bile acid metabolism. Certain bacteria, such as *Clostridium* cluster XIVa, synthesize hydrophobic bile acids (e.g., glycochenodeoxycholic acid, glycoursodeoxycholic acid) [94]. These metabolites induce endothelial dysfunction and promote atherosclerosis, exerting cardiotoxic effects. Several studies have linked increased *Clostridium* abundance with endothelial injury, elevated inflammatory markers (IL-6, endothelin-1, von Willebrand factor), and atherosclerosis progression [44].

In this context, SGLT2 inhibitors improve gut microbiota composition by suppressing TMA-producing bacteria such as *Klebsiella* and *Shigella*. At the same time, they increase the abundance of short-chain fatty acid (SCFA)-producing probiotics like *Butyricicoccus* and *Bifidobacterium*, thereby reducing TMAO production [42]. For example, animal studies show that dapagliflozin can reduce myocardial ischemia-reperfusion injury in diabetic rats by modulating gut-derived TMAO, and lower ferroptosis-related damage [95].

Additionally, SGLT2i remodel the gut bile acid profile to further alleviate cardiovascular toxicity [45]. In animal models treated with dapagliflozin, the overactivation of primary bile acid synthesis is corrected. Total bile acid levels decrease, while hydrophilic bile acids such as glycine-conjugated ursodeoxycholic acid increase and hydrophobic bile acids decrease [40,45]. Clinical studies also link this bile acid profile shift with a reduction in *Clostridium* cluster XIVa abundance [96,97,98]. This change helps improve endothelial dysfunction and slow atherosclerosis progression. The inhibitory effect of SGLT2i on *Clostridium* cluster XIVa has been confirmed in two independent studies [44]. The decline in this bacteria correlates with improvements in blood glucose, LDL-C, and inflammatory markers, suggesting it may underlie some cardiovascular benefits of SGLT2i. Mechanistically, *Clostridium* cluster XIVa has strong adhesion and pro-inflammatory properties, disrupting gut barrier and inducing endoplasmic reticulum stress. Its reduction helps mitigate gut inflammation and endotoxemia [99].

### 5.2. Kidney-Heart Axis: Renal Contributions to Cardiac Injury and the Modulating Effect of SGLT2i

Renal dysfunction is not only a hallmark of chronic disease but also a key driver of cardiac remodeling. Renal decline can directly or indirectly induce cardiac injury through multiple pathological mechanisms, forming the so-called kidney-heart axis.

Firstly, decreased glomerular filtration leads to sodium and water retention, increasing volume load. This elevates ventricular wall tension and chamber dilation, which over time induce myocardial hypertrophy, septal thickening, and cardiac remodeling. Persistent volume overload also raises pulmonary circulation pressure, causing pulmonary hypertension and right heart strain, ultimately leading to heart failure with preserved ejection fraction (HFpEF) or reduced ejection fraction (HFrEF). These kidney-originated effects are well-established clinical targets [100,101,102]. Secondly, impaired renal toxin clearance results in accumulation of microbial and metabolic toxins, creating a vicious cycle that amplifies toxin-induced cardiac damage. This promotes cardiovascular injury and drives left ventricular remodeling and functional decline. Even early CKD increases myocardial injury risk. Studies link mild renal dysfunction (eGFR < 90 mL/min/1.73 m^2^) to elevated heart failure biomarkers such as NT-proBNP and hs-CRP. Moreover, CKD patients often have anemia, secondary hyperparathyroidism, inflammation, and sympathetic activation, which together increase cardiac workload and accelerate heart failure progression [103].

SGLT2 inhibitors exhibit unique potential to modulate the pathological feedback of the kidney-heart axis through systemic actions. Firstly, they improve renal function and structural stability, significantly reducing cardiac volume and metabolic burden. These drugs lower glomerular hyperfiltration and hyperperfusion, reduce proteinuria, and delay renal decline. They promote sodium-glucose cotransport excretion, producing a “selective diuresis” effect. This alleviates volume overload, reduces cardiac preload and afterload, and provides a favorable hemodynamic basis for ventricular remodeling. Such kidney-heart protective effects have been recognized by KDIGO and ESC, and incorporated into standard CKD treatment guidelines [104,105]. Secondly, improved renal function enhances toxin clearance, lowering microbial-derived cardiotoxic substances such as TMAO, IS, and PCS. This reduces their harmful effects on vascular endothelium, cardiomyocytes, and inflammatory pathways. By decreasing systemic toxic metabolite burden, SGLT2i disrupt the core pathological chain of “toxin retention–inflammation activation–ventricular remodeling” in the kidney-heart axis. Moreover, SGLT2i optimize gut microbiota composition and metabolic activity, reducing intestinal toxin generation. This mechanism indirectly reduces cardiac toxic exposure via the gut-kidney-heart axis, providing a dual protective barrier for the heart. Ultimately, these combined effects improve cardiac function. Clinical trials such as DAPA-HF and EMPEROR-Reduced have confirmed that SGLT2i significantly improve left ventricular ejection fraction (LVEF), reduce heart failure hospitalizations, and lower all-cause mortality [106,107].

In conclusion, SGLT2 inhibitors interrupt the pathological kidney-heart feedback via kidney protection, toxin mitigation, and volume regulation. They also coordinate gut metabolic improvements, jointly alleviating cardiac remodeling and functional decline. This establishes a systemic protective barrier from kidney to heart, offering new perspectives for heart failure prevention and treatment.

### 5.3. Direct Cardiac Effects of SGLT2 Inhibitors

Although cardiomyocytes express little or no SGLT2, recent studies have shown that SGLT2 inhibitors, especially empagliflozin, can directly protect the heart independent of their systemic metabolic or renal effects. One important mechanism is the inhibition of the Na^+^/H^+^ exchanger 1 (NHE1), which reduces intracellular Na^+^ and Ca^2+^ overload, prevents abnormal autophagy, and helps maintain cardiomyocyte viability [108,109]. In addition, SGLT2 inhibitors can activate the AMPK/mTOR pathway to enhance autophagy and mitophagy, restoring normal autophagic flux and protecting the heart from remodeling in models of myocardial infarction and HFpEF [110,111]. In models of metabolic stress or Parkinson’s disease-related cardiac injury, FUNDC1-dependent mitophagy has been shown to preserve mitochondrial integrity and reduce apoptosis [112]. Empagliflozin also improves mitochondrial energy metabolism by increasing ATP production, stabilizing the mitochondrial membrane potential, and promoting ketone utilization, which together enhance cardiac energy efficiency under ischemic or stress conditions [113]. Other pathways, including ErbB4- and SIRT3-mediated autophagy, further help reduce cardiac hypertrophy and fibrosis [114].

Overall, these findings suggest that SGLT2 inhibitors not only protect the heart systemically by interrupting the pathological gut-kidney-heart feedback loop, but also act directly on cardiomyocytes to regulate ion balance, autophagy/mitophagy, mitochondrial function, and stress signaling. Together, the systemic and direct effects complement each other, improving cardiac structure and function and ultimately supporting better patient outcomes.

## 6. Integrated Multi-Organ Mechanisms of SGLT2 Inhibitors

Although the gut, kidney, and heart axes offer distinct mechanistic insights, growing evidence shows that the effects of SGLT2 inhibitors (SGLT2i) extend beyond any single organ or pathway. Instead, they operate within a tightly connected multi-organ network, where gut microbial ecology, renal hemodynamics and metabolism, and myocardial energy homeostasis interact and regulate each other, producing clear cross-organ synergy.

SGLT2i remodel the gut microbiota and strengthen the mucosal barrier, reducing gut-derived inflammatory mediators and toxic metabolites, which eases the burden on the kidney and heart. Improved renal function suppresses systemic neurohormonal activation, lowering cardiac stress and oxidative damage. Stabilized myocardial energy use further reduces harmful feedback to the kidney and gut. Together, these effects form a protective framework of reduced inflammation, metabolic stability, and weakened inter-organ crosstalk.

**Figure 2:**
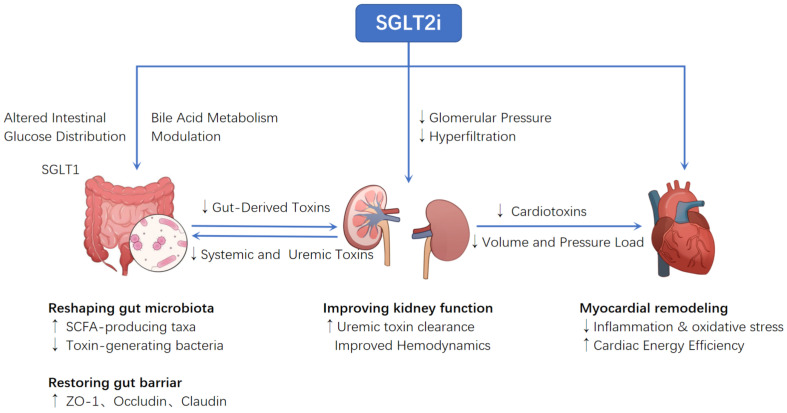
SGLT2 inhibitors improve gut microbiota, renal function and cardiac remodeling by regulating intestinal glucose distribution, bile acid metabolism and glomerular pressure. They reduce toxin production while enhancing energy efficiency and barrier function.

From this integrated perspective, SGLT2i act more as a systemic recalibration than a local fix. By disrupting the gut-kidney-heart vicious cycle, they shift the body from chronic inflammation and metabolic imbalance toward a more stable, lower-load state. The multiple benefits of SGLT2i thus reflect coordinated improvements across the organ network, rather than a single mechanism (Figure 2).

## 7. Conclusions

This review uses the gut-kidney-heart axis as a unifying lens to reconsider how SGLT2 inhibitors may offer protection across organs. Current evidence suggests that their benefits reach far beyond glucose control. SGLT2 inhibitors may help stabilize the gut microbiome, lower the burden of gut-derived toxins, ease renal hemodynamic and inflammatory stress, and support more efficient energy use in the heart. When these changes occur together, they may weaken the pathological loop linking the gut, kidney, and heart, creating a broader shift toward a more balanced physiological state.

Even so, the mechanisms we have today do not fully explain the consistent cardiorenal benefits seen in clinical trials. Many proposed pathways are based on animal or cell studies, and the differences between experimental models and human biology mean that these findings likely reflect only part of the whole picture. At this stage, the evidence points to a series of possible mechanisms rather than a confirmed causal model. More human-based work will be needed to understand how these pathways interact and which of them matter most in real clinical settings. It is worth noting that, despite these gaps, the clinical outcomes associated with SGLT2 inhibitors are strong and reproducible, suggesting that the true mechanism may be broader and more integrated than what is currently known.

Looking ahead, several research directions appear essential. Longitudinal human cohorts and multi-omics approaches may help map the shifting relationships among the microbiome, metabolites, kidney function, and cardiac responses during SGLT2 inhibitor therapy. Causal inference tools and new experimental platforms—such as organoids and spatial transcriptomics—could help pinpoint key regulatory nodes and reveal whether different SGLT2 inhibitors act through distinct patterns. As evidence accumulates, we may move from a general framework toward a clearer and more mechanistic understanding, laying the groundwork for future therapies that target multiple organs in a coordinated way.

## Data Availability

Data sharing is not applicable since no new data were generated.

## Abbreviations

The following abbreviations are used in this manuscript:

AMI	Acute Myocardial Infarction
BA	Bile Acid
CKD	Chronic Kidney Disease
eGFR	Estimated Glomerular Filtration Rate
FMI	Fecal Microbiota Transplantation
GLP-1	Glucagon-Like Peptide-1
HF	Heart Failure
LPS	Lipopolysaccharide
SCFA	Short-Chain Fatty Acid
SGLT2i	Sodium-Glucose Cotransporter 2 Inhibitors
TMA	Trimethylamine
TMAO	Trimethylamine-*N*-oxide
ZO-1	Zonula Occludens-1
